# “The Cone of Shame”: Welfare Implications of Elizabethan Collar Use on Dogs and Cats as Reported by their Owners

**DOI:** 10.3390/ani10020333

**Published:** 2020-02-20

**Authors:** Yustina Shenoda, Michael P. Ward, Dorothy McKeegan, Anne Fawcett

**Affiliations:** 1 Sydney School of Veterinary Science, University of Sydney, Camperdown, NSW 2006, Australia; yshe0403@uni.sydney.edu.au; 2 Sydney School of Veterinary Science, University of Sydney, Werombi Road Camden, NSW 2570, Australia; michael.ward@sydney.edu.au; 3 Institute of Biodiversity, Animal Health and Comparative Medicine, College of Medical, Veterinary & Life Sciences, University of Glasgow, Bearsden Road, Glasgow G61 1QH, UK; Dorothy.McKeegan@glasgow.ac.uk

**Keywords:** Elizabethan collar, quality of life (QOL), animal behaviour, animal welfare

## Abstract

**Simple Summary:**

Elizabethan collars are routinely used in veterinary medicine as a non-pharmaceutical measure to prevent self-trauma and protect certain sites on the body, limbs and head of dogs and cats, and associated negative welfare states. Despite their regular use, very little is known about the welfare implications of these collars. An online survey, aimed at owners whose pets wore an Elizabethan collar during the past 12 months, was utilised to investigate the impact that these collars had on their animal’s quality of life. We found that the majority of the 434 participants (77.4%) reported a poorer quality of life in their companion animals while the collar was worn, based on effects in a range of welfare domains including nutrition, environment, health, behaviour and mental state. A poorer owner-perceived quality of life score was more likely when the Elizabethan collar interfered with the animal’s ability to drink, play or caused irritation. Our findings characterise the specific welfare impacts of Elizabethan collar use, and lead to a recommendation for improved owner awareness of possible harms and the use of alternatives where possible.

**Abstract:**

Elizabethan collars are used in companion animals primarily to prevent self-trauma and associated negative welfare states in animals. However, they have been anecdotally associated with negative impacts on animal health and welfare including distress, abraded/ulcerated skin and misadventure. This study aimed to characterise the welfare impacts of Elizabethan collar use on companion dogs and cats, as reported by owners. Owners of pets who wore an Elizabethan collar during the past 12 months were surveyed about the impacts that the use of Elizabethan collars had on animal activities, in particular sleep, eating, drinking, exercise, interactions with other animals, as well as overall quality of life (QOL). The majority of 434 respondents (77.4%) reported a worse QOL score when their companion animal was wearing the collar, significantly so when the Elizabethan collar irritated their pet or impacted on their ability to drink or play. While other factors are likely to impact animal welfare during veterinary treatment that necessitates the use of Elizabethan collars, this study suggests that Elizabethan collars themselves might have negative welfare impacts in a range of domains including nutrition, environment, health, behaviour and mental state. We recommend that animal owners are informed about potential negative impacts of Elizabethan collars and harm minimisation strategies. Where possible, alternative methods of preventing self-trauma should be explored.

## 1. Introduction

**Figure 1:**
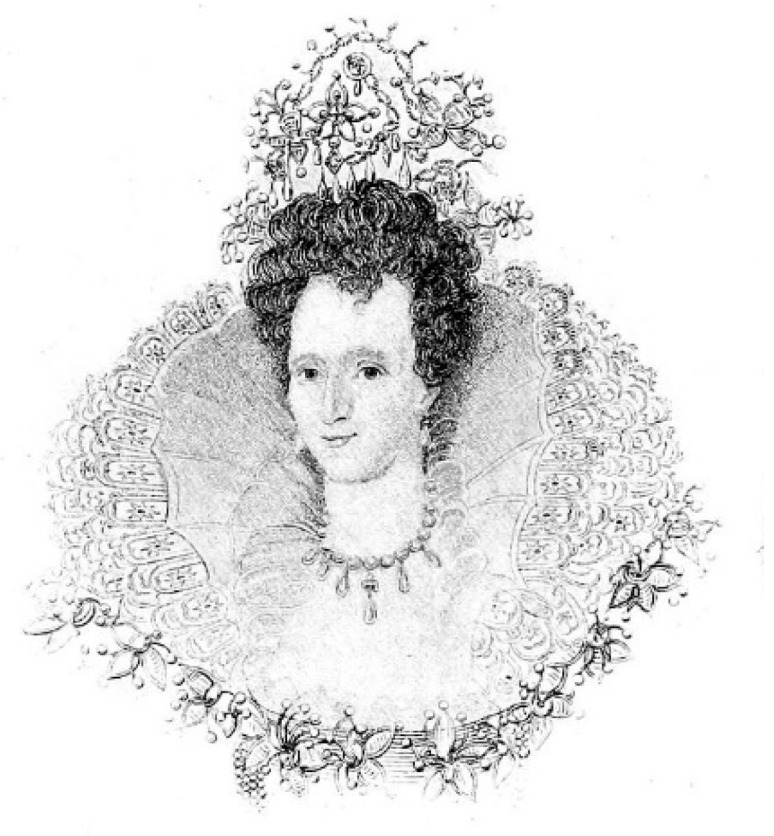
Elizabethan collars are named after lace collars or ruffs worn during the era of Queen Elizabeth I, as illustrated in this portrait of Queen Elizabeth (source: https://commons.wikimedia.org/wiki/File:Two_Portraits_of_Queen_Elizabeth,_Illustrating_Wide_Ruff_and_Headress-_Elizabethan_People_(book).jpg; https://archive.org/details/cu31924027958630).

Elizabethan collars are routinely recommended by veterinarians, most commonly to prevent dogs and cats from removing their sutures following surgery. Elizabethan collars are designed to prevent the animal from reaching their body with their mouths and to protect the head, eyes, face and neck from being scratched by the limbs [1,2,3,4]. They may also be used in other companion animal species such as birds, small mammals and rodents [1,5,6]. They are named after lace collars or ruffs that were fashionable at the time of Queen Elizabeth I [1] (see Figure 1).

**Figure 2:**
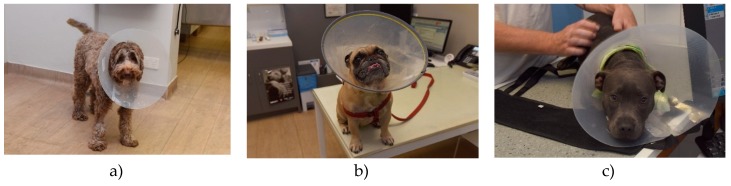
(**a–c**): Dogs wearing commercially available, adjustable rigid plastic Elizabethan collars to protect surgical wounds.

**Figure 3:**
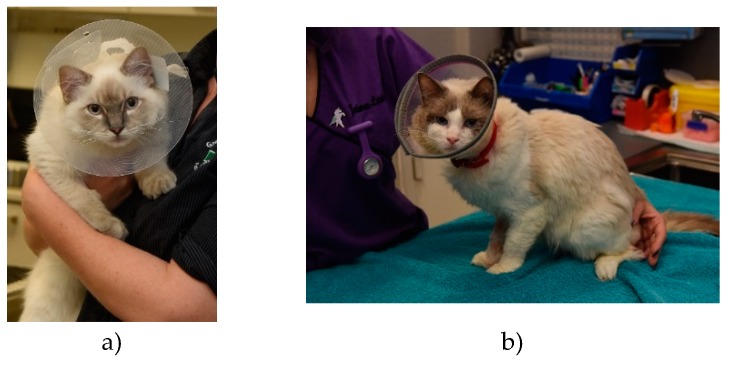
(**a,b**): Cats wearing commercially available, adjustable rigid plastic Elizabethan collars to protect surgical wounds.

**Figure 4:**
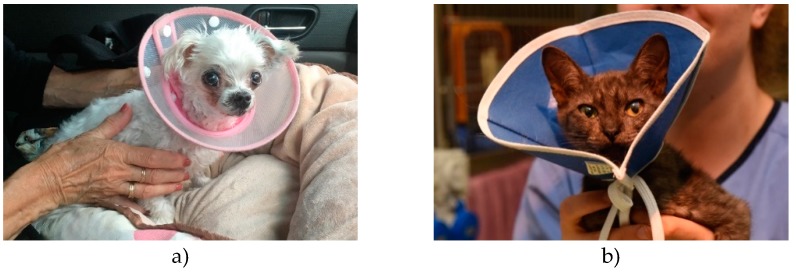
A dog wearing a rigid plastic Elizabethan collar with a soft inner flange and stud-buttons for quicker and more secure fitting; (**a**) A cat wearing a collar made of flexible plastic material (**b**).

References of their use in veterinary medicine date at least as far back as 1897 [7], with an earlier source referring to them as ‘puzzles’ [8]. Like ruffs worn at the time of Queen Elizabeth, these devices have both an inner (around the neck) and an outer flange (rim of collar) and the funnel opening can be directed cranially or caudally [1]. Earlier Elizabethan collars for companion animals (circa 1906) were made of wood, leather or steel [7]; some sources described the application of buckets with a hole in the bottom on dogs to prevent self-trauma [9]. Variations on the design, including features to facilitate adjustment of the diameter or the addition of padding over the outer flange, are available to ensure collars achieve their purpose and/or to improve comfort [10]. Currently, commercially available veterinary Elizabethan collars are predominantly made from rigid plastic (see Figure 2a–c, and Figure 3a,b), although softer plastic collars are available (see Figure 4a,b) [1,2,3,5,11]. Elizabethan collars come in different sizes, which are selected according to the animal’s size, conformation, temperament and the location of the site requiring protection [5]. 

Elizabethan collars may be modified to improve comfort and reduce the risk of injury to animals. Examples include Velcro or stud-button fastening for quicker and more secure fitting, padded banding around edges to reduce irritation, and flexible material which is useful when the animal lies down [10] (see Figure 4a,b). 

There are many valid reasons why collars are utilised. When dogs or cats experience pain or pruritis they may lick, bite, chew or scratch the area [12,13]. Self-trauma can cause inflammation, abrasion or excoriation, and potential secondary infection which may contribute to pruritis [10]. Self-trauma, secondary infection or a combination of these can lead to the breakdown of surgical wounds [1,2]. Elizabethan collars are primarily used in veterinary clinical settings post-operatively to prevent suture removal or as a non-pharmaceutical method of preventing self-mutilation or self trauma [1,3,11]. In these settings, they may also be used to avoid removal of external devices such as catheters and bandages [1]. Their application has also been reported in pseudopregnant (physical state presenting signs reflecting that of a pregnancy, without the presence of a foetus) dogs to prevent stimulation of lactation via auto-stimulation of the mammary glands [14,15]. They may be used to break the itch–lick cycle in animals with pruritic skin disease [13]. Elizabethan collars have been used to protect ECG recording equipment worn by dogs in experimental settings [16]. Moreover, the use of Elizabethan collars has been reported to control hyper-auto oral grooming in cats [17].

However, there are reports that Elizabethan collars may be perceived by companion animal owners as cumbersome, and that many owners are reluctant to maintain Elizabethan collars on their animals for the duration required [13]. Additionally, collars may fail, with animals still able to access the site that the Elizabethan collar is being used to protect [13].

Owner reluctance to maintain Elizabethan collars may be due to changes in animal behaviour when Elizabethan collars are worn, and because they can potentially have a negative impact on animal welfare. In relation to animals in laboratory settings, Brown suggested that Elizabethan collars can increase stress levels, result in abraded or ulcerated skin around the neck as well as lead to aggressive interactions with other animals [1,5]. Assisted feeding may be necessary as many pets struggle to eat and drink or may have a depressed feed intake while wearing the collar [1,2,5]. Additionally, Elizabethan collars generally result in a narrowed field of view, have the potential to obstruct peripheral vision and hearing and may be frightening and uncomfortable for dogs and cats [11]. The prolonged use of Elizabethan collars in cats may result in increased flea burden due to interference with the animal’s ability to groom [18]. Some animals may experience allergic reactions to the plastic used to make the collar [19].

Additionally, Elizabethan collars may cause harm to animals wearing them. Wilson (1993) reported two cases of asphyxiation in dogs wearing Elizabethan collars who became caught in plastic bags [20]. Ill-fitted collars and inadequate monitoring can result in injuries for pets, possible deaths, as well as further costs for their owners and liability to veterinarians [2,5,20]. Animals can potentially damage the collars through means of scratching, clawing or chewing the collar itself, therefore rendering the device useless and potentially hazardous if there are sharp edges [1,2,3,4]. 

Despite these reported drawbacks, such devices are routinely used in veterinary medicine and animals may be discharged from clinics wearing an Elizabethan collar as a part of their treatment plan [1,2]. There is recent concern that routine veterinary care may contribute to fear, anxiety and distress in companion animal patients [21]. As Elizabethan collars may be a component of routine veterinary care, it is critical for animal health professionals to understand the potential for iatrogenic harm associated with these devices.

Although Elizabethan collars have been associated with a range of potential welfare issues, there has been no attempt to formally characterise and quantify the severity and likelihood of these harms. We therefore sought to determine the impact of Elizabethan collars on canine and feline welfare and the owner’s perception of their quality of life (QOL). This was assessed through use of an online survey directed at owners whose dogs or cats had worn an Elizabethan collar in the past twelve months. 

## 2. Materials and Methods 

### 2.1. Survey Tool

The questionnaire consisted of 21 to 27 questions, depending on the respondent’s answers (see Appendix A.1). Five questions related to participant demographics (respondent’s age, gender, postcode, whether they were a veterinarian, veterinary nurse, veterinary technician, animal trainer or person who works with animals for a living, and to what degree they were responsible for the animal’s care in the household). 

The remaining questions (16–22 items) focused on Elizabethan collars, including reason for wearing; duration of use; impact on a range of activities on a score of 1 to 5 (1 = cannot perform this activity when wearing collar; 2 = has some difficulty performing this activity when wearing collar; 3 = needs assistance when performing this activity; 4 = Elizabethan collar makes minimal difference when performing this activity; 5 = Elizabethan collar makes no difference to performance of this activity); whether owners were able to check whether the Elizabethan collar was fitted appropriately; whether the animal experienced any breathing difficulties with or without the collar on; whether the Elizabethan collar needed to be replaced; whether the animal sustained any injuries related to the collar; whether the animal’s response to sound changed while wearing the collar; whether the animal or owner removed the collar; whether alternatives to the collar were used; and whether interactions between the animal and other animals in the household changed during the period in which the collar was worn. Respondents were also asked to rate their companion animal’s overall quality of life (QOL) with and without the Elizabethan collar, on a scale of 1–5, where 1 indicated QOL which “couldn’t be better” and 5 indicated QOL which was “very poor”. Respondents were asked whether they wished to provide any additional comments and were provided a free-text field to do so.

Research Electronic Data Capture (REDCap) was the survey platform used. REDCap is a secure web application hosted by the University of Sydney that can be used for building and managing surveys, as well as data storage and export [22]. The study was approved by the University of Sydney Human Research Ethics Committee (HREC) (project 2019/064).

### 2.2. Recruitment 

The survey was piloted by veterinarians and veterinary students. Questions were refined and the final survey was open from 4th of March to the 1st of May 2019. The link was shared via University of Sydney social media accounts including Facebook and Twitter. Followers of these accounts were able to share the link if they wished to. The link was also shared in News Limited local newspapers (syndicated in NSW, QLD and Victoria), and shared in the United Kingdom (UK) by Dr. Dorothy McKeegan (Senior Lecturer at the University of Glasgow).

To meet the inclusion criteria for the survey, respondents were required to have had a dog or cat who wore an Elizabethan collar during the previous 12 months. Participation was open to all geographic locations.

### 2.3. Statistical Analysis 

Responses were exported from REDCap to Excel and analysed using IBM® SPSS® statistics v. 24 (IBM, Armonk, NY). Data were checked for errors such as non-logical values and missing values were noted. QOL scores (1 ‘couldn’t be better’ to 5 ‘very poor’) of companion animals before and while wearing the collar were compared and outcome categories were generated based on the difference (better, neutral, worse). Positive QOL scores (+1, +2, +3, +4) were categorised as ‘better’, negative QOL scores (−1, −2, −3, −4) as ‘worse’ and scores of ‘0’ as ‘neutral’. Predictor variables were then screened for association with this outcome (better, neutral, worse QOL score) using chi-squared tests (SPSS v. 24). Responses ‘don’t know’, ‘not sure’ and ‘can’t recall’ were treated as missing for data analysis. If necessary, some variable categories were collapsed to meet assumptions of the chi-squared test. Variables associated with the outcome at *p* < 0.2 were then included within a forward stepwise, ordinary logistic regression analysis, in which the outcome was re-categorised to be binary: worse (1) versus neutral or better (0) QOL score difference. The likelihood ratio test was used to select variables. The final model fit was assessed using a Hosmer–Lemeshow test and evaluated using Nagelkerke r^2^ statistic. Risk was interpreted using exponentiated model coefficients (i.e., odds ratios).

### 2.4. Thematic Analysis

Additional comments were uploaded into NVivo Pro 11 qualitative and mixed methods software. Thematic analysis was performed as outlined by Braun and Clarke [23,24]. Briefly, two authors (YS and AF) read through each additional comment. Each comment was then coded inductively for semantic themes, employing a realist approach without a pre-existing theoretical framework. An iterative approach was used, beginning with assigning codes to each comment. The same comment could have several different codes assigned, so that any comment could remain uncoded, be coded once, or be coded multiple times. After initial coding, the list of codes was examined to identify clusters of codes which were grouped together and identified as themes. Themes were reviewed for both internal coherence and distinctiveness from other themes. This involved reading all coded extracts from each theme. Where comments did not fit a theme, these were either reallocated to a more appropriate theme, allocated to a new theme, or discarded from the analysis.

The validity of individual themes was then considered in relation to the entire dataset, and, where necessary, additional comments were coded within established themes. Representative comments were selected for use in the text. Where respondents specified that they were dog, cat or dog and cat owners, we included this information. Where respondents did not specify the species of animal(s) they owned, we attributed such comments as unspecified.

## 3. Results

A total of 434 participants completed the survey; of these, 93.5% were female (406/434), 5.1% male (22/434) and 0.7% other (3/434) (Table 1). Survey respondents self-selected to participate and hence a response rate cannot be calculated. The majority (66.6%) of respondents were from Australia (289/434) with the remainder (29.3%) of responses from the UK, USA, NZ, South Africa, Ireland and Sweden. Some (4.1%) of the respondents did not provide their postcode. Respondent age ranged from 18−84 years old, the average age was 40. Most (24.9%) participants were aged between 30 and 39 years old and 0.9% of participants did not provide information about their age. Neither the age (Mann−Whitney U-test *p* = 0.960) nor the sex (chi-square test statistic 1.97, df 1, *p* = 0.160) distribution of respondents from Australia versus oversea were significantly different.

The majority (63.6%) of survey participants did not work with animals for a living (e.g., veterinarian, veterinary nurse, veterinary technician or animal trainer). The majority (71.4%) of respondents (310/434) were the primary person responsible for the care of their pet, 27.9% (121/434) shared the responsibility between household members and 0.7% (3/434) said that it was mostly someone else’s responsibility. 

Most (36.4%) owners reported that their animal wore the Elizabethan collar for a duration ranging from 72 h to 7 days. However, in free-text responses, participants indicated that some animals wore them continuously for periods of 6 months, or intermittently for years (Table A1). 

The majority (57.4%) of animals were required to wear the Elizabethan collar to protect a surgical site on the body, or to prevent self-trauma because of a skin condition (19.1%). Other reasons reported included preventing self-trauma due to anxiety, to facilitate administration of ophthalmic medications, and, in one case, to protect an owner from being bitten when they administered insulin injections to a diabetic, “needle-phobic” dog.

Overall, Elizabethan collars reportedly interfered with several everyday activities for the majority of animals wearing them, particularly eating, drinking and playing. The majority (60.2%) of owners reported that their pet demonstrated difficulties drinking while wearing the collar and 17.1% said that their pet could not perform this activity at all while the collar was on. Two-thirds (67.5%) of respondents reported that the Elizabethan collar interfered with their companion animal’s ability to play. Degree of interference with playing included not being able to play at all (19.1%), having trouble playing (28.8%) or needing assistance to play (19.6%). Additionally, 10.4% reported that the Elizabethan collar interfered with “other” activities. Activities listed included toileting, cleaning themselves after toileting, self-grooming, being fitted for a harness or lead, getting through the dog or cat door, sleeping in a crate, navigating stairs or moving around indoors “without smashing into doorways, tables or chairs”. The Elizabethan collar appeared to have less of an impact on walking, other exercise, interacting with people, resting, sleeping and outdoor access. 

The vast majority of respondents (88.0%) reported they were aware of how to check whether the Elizabethan collar was too tight/loose. Almost all respondents (98.2%) reported that their animal did not exhibit any difficulty breathing while wearing the Elizabethan collar.

The majority of respondents (70.5%) did not need to re-size or replace the collar during the time it was worn. However, 28.1% of respondents did. Reasons for replacing the collar included wear and tear of the collar, physical damage to the collar, soiling of the collar from food and/or wound discharge, changing to an alternative (for example from rigid plastic to inflatable, or vice-versa), and a need to re-size the collar. Several owners reported that the right size collar was not available from their veterinarian or other source (for example, “both sizes were not right but no other sizes”), while a number realised the collar was not appropriately fitted when it was removed by the animal, or when the animal was able to access the site(s) the collar was being used to protect in spite of the Elizabethan collar (for example “she figured out how to lick herself around it”). 

Approximately 25% of respondents reported that their animal had experienced an Elizabethan collar related injury. Of respondents who noted injury to their companion animals as a result of wearing the collar, the most reported (61.1%) injury was itching/irritation, followed by “other” (23.9%). Other injuries included trauma due to bumping into walls or objects or falling downstairs, and psychological distress. One respondent reported that their companion animal “developed a yeast infection around the mouth from saliva pooling inside the collar”. Most injuries were minor.

Most owners did not report any change in their animal’s responsiveness to sound. Only 0.2% responded that their animal was not responsive to sound at all while wearing the Elizabethan collar, while 11.5% reported that their animal was less responsive to sound and 19.4% responded that their animal was more responsive to sound.

More than half (54.1%) of respondents removed the Elizabethan collar only for periods that the animal was under supervision, and 24.9% only removed the Elizabethan collar for certain activities, for example when the animal was being fed or given water. Most animals (68.9%) did not remove their Elizabethan collar without assistance, though 23.7% of animals sometimes removed the Elizabethan collar themselves and 4.8% did so frequently. 

Approximately one third (33.6%) of respondents reported that the Elizabethan collar was associated with reduced interaction between the animal and other animals in the household, while 8.8% of respondents reported increased unfriendly interactions between the animal and other animals in the household.

Just over half of respondents reported utilising an alternative to the Elizabethan collar to achieve the same purpose, with the most common being inflatable collars (27.6%), t-shirt or wrap (24.2%), local dressing (17.5%) or other (9.4%). Examples of “other” alternatives used included commercially produced visors, cones or boots, socks, leggings, muzzles, topical bitterants, a customised collar fashioned from a pool noodle, towels, bandages, medication, and positive reinforcement training. Some owners provided constant supervision (for example, one owner reported “just sitting with dog and physically preventing licking the wound”) or physical restraint (“holding him”).

When comparing QOL scores of companion animals before and while wearing the collar, the vast majority (77.4%) of respondents reported a worse QOL score with the Elizabethan collar, some (12.9%) reported a better QOL score with the collar and the remainder (9.7%) reported that there was no difference (neutral). 

Chi-squared tests (SPSS v. 24) were used to screen predictor variables (survey questions excluding demographic information) for an association with the outcome (better, neutral or worse QOL score). Screening of 28 variables identified 15 exploratory variables (*p* < 0.2) (Table A2). 

Significant variables were included within a forwards stepwise logistic regression analysis, in which the binary outcome was re-categorised as worse (1) versus neutral or better (0) QOL scores. Stepwise logistic regression modelling identified ‘drinking’, ‘playing’ and ‘injury—itching/irritation’ factors as being the best predictors of a ‘worse’ QOL outcome (Table 2) (Hosmer and Lemeshow chi-square test statistic 3.770, df 8, *p* = 0.877, Nagelkerke R2 value 0.356). 

Owners who reported that they did not know how to check collar size (12.0%) were not at a high risk for reporting itching/irritation as a result of the collar. 

**Figure 5:**
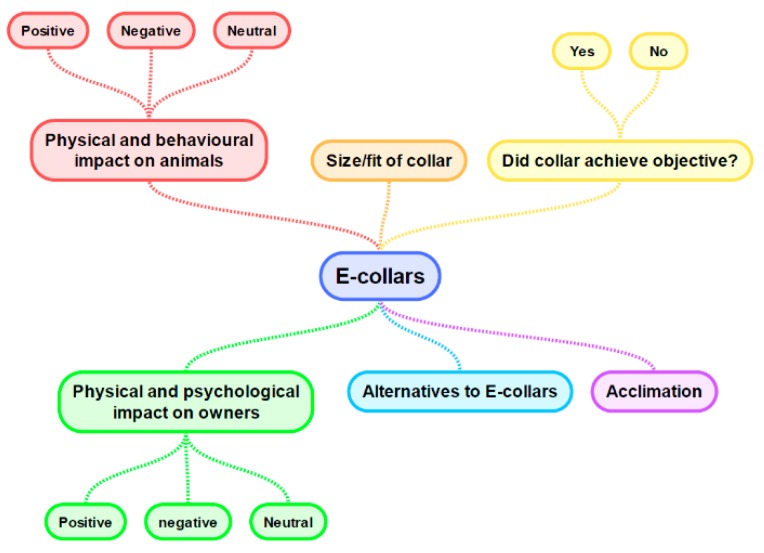
Map of key themes identified via thematic analysis of “additional comments”.

There were 182 additional comments submitted. These were coded into six themes (see Figure 5), as discussed below. 

### 3.1. Physical and Behavioural Impact of Elizabethan Collars on Dogs and Cats

Owners reported that Elizabethan collars impacted companion animals physically, by inhibiting normal behaviour or making it challenging for animals to navigate their home environments. Some animals appeared to overcome these difficulties, with owners often praising their animal’s problem-solving skills.

“The collar made him less mobile, which was sad for him but made it much easier for me to find him, get hold of him and apply the various eye treatments.”

—cat owner.

“Our cat just stood in a crouched still position while wearing the Elizabethan collar.”

—cat owner.

“The collar made navigating around furniture difficult for the dog as the edge would catch on objects or on my legs.”

—dog owner.

“I have a dog door and when he first came home he was obviously confused as to why he could not get through the opening. It took him probably one day to work out that if he turned his head sideways and squished the collar he could get in and out.”

—dog owner.

Others required assistance, particularly with eating and drinking.

“She refused to eat or drink for 2 days. We had to hand feed her and wet her mouth. We couldn’t take the collar off without hurting her so it had to stay on the whole 4 weeks until the stitches came out.”

—dog owner.

“We have raised food dishes for our dogs, so if we fed in a bowl she could access her food while wearing the e collar but she could not eat out of her Kong or other food dispensing toys.”

—dog owner.

Owners reported that their animals experienced psychological distress due to the presence of the Elizabethan collar, and seemed much happier without it.

“…unfortunately his mood was very low with it on”

—dog owner.

“The collar made her life miserable. Since she is terminal, I didn’t want her last days to be awful and uncomfortable.”

—dog owner.

“I noticed that my cat walked lower and dragged the collar along the wall. She seemed really depressed. Each day I’d go to work and I’d come home and she’d managed to remove it and she was happy about that. Once it stayed off she was back to her old self.”

—cat owner.

Some owners perceived that their animal’s stress associated with wearing the Elizabethan collar was a bigger welfare issue than the problem the Elizabethan collar was intended to address.

“His distress with this collar was worse than his constant licking.”

—unspecified.

“I took it off because he was so stressed and had to attend vet because he would not urinate due to stress”

—unspecified.

“my female cat became immediately distressed as we brought her home with the collar on and frantically ran around the house smashing into things.”

—cat owner.

Owners reported that animals wearing Elizabethan collars were prone to misadventure, frequently describing a tendency to walk, run or otherwise bump into objects or people, or an increased frequency of falling.

“My cat kept walking backwards and falling over so became dangerous when on the bench etc. Was very stressed. I caught her a few times about to fall.”

—cat owner.

“Running in to people, furniture and doorways was the biggest obstacle.”

—unspecified.

One owner described the challenge presented by heavy snowfall:

“…in our case it was 22’ of snow that made it impossible for him to move outside without have the snow fill the collar”

—dog owner.

A number of owners speculated that Elizabethan collars inhibited the vision or hearing of their companion animals, though whether this was considered negative, neutral or positive appeared to depend on the animal’s circumstances and temperament.

“The collar combined with his eye injury seemed to make him more sensitive to sound and movement near him which he couldn’t see properly because of his eye injury and because of the collar.”

—cat owner.

“My dog despised the Elizabethan collar as it makes it difficult for her to hear properly, especially with identifying where the sound is coming from. It greatly disorients her”

—dog owner.

“On this occasion, the Elizabethan collar was on a young foster dog who had been rescued from a regional pound and was absolutely terrified when we started fostering her. She was desexed about 2 weeks after she arrived and I noticed that her behaviour became more confident while she was wearing the Elizabethan collar. I’m not sure if this was because sounds were blocked or muted or it gave her a feeling of more space around her which made her feel more secure.”

—dog owner.

The collar had a variable impact on interactions between the animal and other companion animals in the household.

“The only issue we had is his sister acting aggressively [sic] toward him due to being scared of the collar this finally settled after a few days.”

—cat owner.

“Dog is deaf, small breed and soft cat Elizabethan collar was used. Made no difference to her normal interactions.”

—dog owner.

“My standard poodle wore the Elizabethan collar for 3 weeks after her spey. Once she got used to it there was no stopping her. She did annoy my other standard though. She would want to play and would ram the Elizabethan collar into the other one. Occasionally my older standard [poodle] would tell her off.”

—dog owner.

“The other dog in the house was scared of Grace while she was wearing her collar and as a result was very unfriendly towards her.”

—dog owner.

“The increased unfriendly interactions from the other animal in the home was not serious or concerning. The other animal was more interested in what that thing was!”

—unspecified.

“One of my dogs is mildly aggressive, and when the other dog wears an Elizabethan collar and bumps her she will growl or snap at him which is not typical.”

—dog owner.

A number of owners reported that their animal had pre-existing anxiety, which was exacerbated by the collar.

“I have a highly anxious dog who needed to wear the collar due to obsessive licking that was causing him injury. Unfortunately, although the collar allowed the time for some wounds to heal, his anxiety was exacerbated during this time and it took a number of days after wearing for him to return to his usual (still anxious) state”

—dog owner.

“The cleaning of the collars is a real pain. My cat has some anxiety anyway, so after the trip to the vet, operation, stay in the vet hospital, coming home but with the cone of shame made settling him and his recovery even more difficult.”

—cat owner.

### 3.2. The Size and Fit of Elizabethan Collars

Owners reported that ill-fitting or inappropriately sized Elizabethan collars made it difficult for dogs to perform activities such as eating and drinking.

“The collar we received after her surgery was too large for her size and as such (size at-large for 55 lbs dog), she couldn’t drink water or eat food as her snout was not long enough to reach and kept tipping the bowl or stopping her from getting water. I could easily slip the collar over her head at the tightest setting (I then secured it to her regular collar).”

—dog owner.

“An Elizabethan collar is a real nuisance for small dogs with short legs. When they drop their head to see where they’re going or to pick something up it also drops and can stick into the ground, pulling them up sharply.”

—dog owner.

A number of owners provided comments related to animal conformation or breed-specific challenges associated with Elizabethan collars:

“Collar was to protect a surgical site. Great Dane means huge collar, he couldn’t cope with it.”

—dog owner.

“…these collars just don’t fit greyhounds. Following our dog’s second injury, an interstate vet who obviously didn’t have much experience with greys sold us a collar that was far too small for her...her nose poked out the end, and when she bowed her head it slid off!”

—dog owner.

“I have a mini dachshund, so the Elizabethan collar had to be quite long to accommodate his longer snout. Because his legs are so little he had a lot of difficulty with the large Elizabethan collar doing lots of things such as eating and getting outside down a small step.”

—dog owner.

“My dog is a bull-dog and his neck got very wet and inflamed from slobbering constantly with it on. He got very down with it on and seemed depressed. Maybe the shape of it was not good for him.”

—dog owner.

“We have sighthounds (greyhound and Saluki). The size of the collar to cover their long noses was absolutely ridiculous. Like wearing an umbrella around the neck. The dog was in misery.”

—dog owner.

### 3.3. Outcomes of Wearing Elizabethan Collars

A number of owners reported that Elizabethan collars, when used appropriately, could benefit animals by eliminating self-trauma, protecting surgical sites and facilitating healing, essentially achieving the purpose they were intended for.

“I have had a number of cats and dogs wearing Elizabethan collars over the years. They have never caused a problem with either cats or dogs. Definitely the best way to stop animals interfering with sutures or vulnerable areas”

—dog and cat owner.

“I think that an Elizabethan collar can be a great tool when used properly. If an animal if sensitive to the collar, other methods can be considered”

—unspecified.

“The collar was very effective in ensuring that the wound healed completely with no licking or scratching at it.”

—cat owner.

“The E collar does its job on protecting my dog from licking her surgery wounds.”

—dog owner.

For some owners the Elizabethan collar was framed as a necessary evil, with the benefits outweighing acknowledged, at times significant, costs:

“Made the cat thoroughly miserable but he had licked himself a huge ulcer and the collar was the only way to get it healed. The collar interfered with virtually all aspects of his life, he hated it but fortunately was too stupid to figure-out how to get it off.”

—cat owner.

“The collar although inconvenient was necessary to avoid removal of dressings and I believe that the positive elements over-powered the negatives”

—unspecified.

“Elizabethan collars are necessary. Doesn’t matter if you or your dog like them it keeps them safe from their selves”

—dog owner.

“Elizabethan collar was essential to stop my dog rubbing even briefly at his eye (entropion and enophthalmos). Far, far better that he didn’t disturb the delicate sutures. I’ve seen several dogs disembowel themselves worrying at their surgical site. All but one was DOA [dead on arrival]. the one that survived lost quite a bit of intestine as she chewed on it.”

—dog owner.

However, some respondents reported that Elizabethan collars did fail to achieve their intended objective, most frequently due to the behaviour of the animals wearing them. A number of owners reported that their companion animals found a way to work around the collar or remove it altogether. In some cases, this necessitated additional veterinary intervention. In other cases, owners had to anticipate their animals’ behaviour to prevent collar removal.

“Dog was still able to tear out stitches in collar. Learned to lean against wall/furniture and flatten it to enable access to wound”

—dog owner.

“Unfortunately my Pekingese had eye surgery and due to her working out how to rub her eye ( that had stiches ) while wearing the Elizabethan collar, she would aim to push her face to the floor where the plastic edges of the collar touched the floor / cushion she’d push harder so the collar would slide a bit further down her tiny neck and she could rub that eye on the floor/ cushion !!! So another operation was needed, I also bought a larger collar cause I needed the plastic edges longer all the way around, now she couldn’t eat or drink while that collar was on, so I would take it on and off while watching the little rascal like a hawk.”

—dog owner.

“It was a trying 10 days as my too clever cat discovered that she could pull off the collar by running under the bed in the room that she was confined in and knocking it off when it hit the underside of the bed frame. We ended up dismantling the bed and having the mattress on the floor for 10 days.”

—cat owner.

### 3.4. Physical and Psychological Impact of Elizabethan Collars on Companion Animal Owners.

While we did not ask specifically about the physical or psychological impact of Elizabethan collars on the animal owners themselves, it became clear that the collars, and their animals’ responses to them, impacted owners in a variety of ways. A number of owners reported that their quality of life was negatively impacted by the Elizabethan collar, impacting their own stress levels, their home environment, and their sleep.

“Her quality of life was ok. Ours was a bit stressful at times.”

—unspecified.

“I dislike the collar a lot. We live in a small house & our dog would constantly be knocking into furniture & us. Nightmare. That is why I would constantly take it off when I was at home supervising her.”

—dog owner.

“Was constantly bashing into things and seemed bewildered. Restless at night - had to take collar off and sleep next to him to let us both rest!”

—unspecified.

Animals wearing Elizabethan collars injured their owners on occasion, mostly by running into the legs of the owner or the collar becoming caught on the owner’s legs.

“Bruises on my legs are common when the dogs have them on, from them running into my legs”

—dog and cat owner.

“Interacting with him was also very difficult, as he would beat your legs with the collar, which could get pretty painful...”

—unspecified.

“It hurts when they slam into your legs…”

—dog owner.

"The collar made navigating around furniture difficult for the dog as the edge would catch on objects or on my legs”

—dog owner.

“The Elizabethan collar was also aversive to me. My shins were so bruised from him banging into me causing less interactions with us.”

—unspecified.

Similarly, numerous owners reported Elizabethan collars as a source of property damage:

“The collar can cause damage to the house (e.g., scratching walls, damaging furniture as they try walk past)”

—unspecified.

“I have sustained a lot of damage to doors and plasterwork over the years as a result of the collars.”

—dog owner.

### 3.5. Habituation

A number of owners reported training their animal (usually dogs) to wear the collar prior to the dog being required to do so, as a means of reducing stress. Some suggested that training may have assisted some animals in better coping with Elizabethan collars.

“This dog is mentally fit and resilient and was shaped to wear collar BEFORE surgery. She was also crated and had been crated trained from puppy-hood.”

—dog owner.

“I also believe people should be taught how to get their dogs accustomed to wearing (taking on and off etc.) an e collar in a fun and positive way during training or at home so when it is needed for real the dog will not be as stressed by it.”

—dog owner.

“I once had a dog who would shred an Elizabethan collar and he was adamant to get it off. I trained a very good ’leave it’ and he was much more compliant and able to rest without using an Elizabethan collar. I think alternatives to an Elizabethan collar and better training could lessen the stress for pets who would traditionally be prescribed an Elizabethan collar.”

—dog owner.

In addition, some owners reported that their animal (again, usually dogs) appeared to habituate to wearing the Elizabethan collar over variable periods of time.

“He had a lot of trouble managing with the collar for the first ten days or so but got the hang of it after that.”

—dog owner.

### 3.6. Alternatives to Elizabethan Collars

Companion animal owners discussed an interest in finding a suitable alternative to the Elizabethan collar, to reduce perceived Elizabethan collar related stress and discomfort and to facilitate normal behaviour as much as possible.

“I found that a squidgy travel pillow securely fixed around my dog’s neck was more effective and much more comfortable than the plastic Elizabethan collar. We live in a hot, humid climate & the plastic Elizabethan collar seemed to trap hot air and flies (not to mention food scraps...) around my dog’s face”

—dog owner.

“My dog didn’t wear the Elizabethan collar for more than 30 minutes because he refused to move and was terrified every time he bumped into something. He went without for a few days while I waited for a BiteNot collar to be delivered”

—dog owner.

“I replaced the plastic Elizabethan collar with an inflatable one, so my dogs could eat and drink. This was impossible with the plastic one.”

—dog owner.

“Love surgical garments and will always try them first! E collars are last resort.”

—unspecified.

“My dog is very anxious and although I had spent a lot of time beforehand trying to get him used to an Elizabethan collar, I was not able to get him comfortable wearing one. After the first time when he freaked out even after the preparation I had done, he wouldn’t let me put it on and would have bitten me so I used a t-shirt tied to a harness instead, which he was fine with.”

—dog owner.

“The collar used was one of the soft blue papery ones, not the hard plastic. Because it’s a softer construction, for my cat I was able to flip it down so that he wore it over his shoulders. Much more comfortable and tolerable as it wasn’t in his vision.”

—cat owner.

Some owners were interested in alternatives but felt there were limitations with these too.

“Alternatives for the plastic Elizabethan collar were both more and less effective—the inflatable let the dog reach a leg wound, the comfy cone was definitely more comfortable for a dog to wear.”

—dog owner.

“Elizabethan collar is preferable for certain situations whereas another type of preventative equipment might be preferable for other situations.”

—dog owner.

“Wish there were a better design/option. Inflatables are nicer, but rarely stay on.”

—dog owner.

“We initially used alternative methods to prevent the dog from accessing the surgical site, e.g., inflatable collar, t-shirt, pool noodle collar, bitterant, due to the discomfort and difficulty in moving generally associated with an Elizabethan collar. Given the location of the wound (desexing) and the length of the dog, however, we eventually resorted to utilising the Elizabethan collar due to the ineffectiveness of the aforementioned methods. While effective, the Elizabethan collar greatly diminished the dog’s quality of life and ability to manoeuvre without assistance and perform normal activities.”

—dog owner.

Others needed to combine strategies to achieve the intended objective:

“ended up having to use combination of basket muzzle, Elizabethan collar, PJs and sedation to keep from taking off cast”

—dog owner.

“Got an inflatable collar and a sheep shirt for her second surgery and it was much better tolerated.”

—unspecified.

“We had to use an Elizabethan collar plus two inflatable donuts to ensure that our very flexible dog couldn’t reach her hot spot”

—dog owner.

Owners who used alternatives to Elizabethan collars also reported occasional failure or misadventure:

“I did use an inflatable collar and my other dog popped it. Whilst the injured dog was wearing it!”

—dog owner.

“Used a soft Elizabeth collar and a plastic, the soft one allowed her to get a paw between the collar and her neck and she almost choked”

—unspecified.

Others were not aware alternatives existed:

“Was unaware of other alternatives (vet only suggested Elizabethan collar).”

—dog owner.

One owner reported frustration on learning that perhaps an Elizabethan collar prescribed by their veterinarian was not necessary:

“It was interesting that our vet insisted our dog needed the collar for his own good whereas many friends did not use the collar on their pets at all when their animals were desexed. I found this confusing and annoying as I would have preferred no collar on our dog as well even though he got used to it. The collar was a hindrance and we humans felt very bad about making him wear it, especially if it turned out that it wasn’t really necessary after all.”

## 4. Discussion

The results of this study show that, while employed to protect animals, Elizabethan collars are not a benign intervention. Elizabethan collar wearing was associated with worse owner-reported quality of life in 77% of dogs and cats. The effects on welfare were wide ranging, and it is useful to relate the reported issues onto the ‘Five Domains’ framework—devised to assess welfare compromise in sentient animals and subsequently revised to allow consideration of positive states [25]. The Five Domains include nutrition (eating and drinking was affected with some animals unable to feed unassisted or refusing to feed); environment (issues with navigation and movement and increased risk of harm); health (may be improved by collar wearing to promote healing but also risk of injury or skin irritation from the collar); behaviour (restriction of several behaviours was reported including positive states such as play) and mental state (collar wearing reported to be was associated with ‘stress’ and depressed mood in the animal by many owner). 

Elizabethan collars were generally well-fitted: 88.0% of respondents were confident that they could determine whether the collar was too tight or too loose, 98.2% reported that the collar did not impair the animal’s breathing and 70.5% reported that there was no need to re-size or replace the collar. Yet they interfered with animals’ daily activities.

Owners were significantly more likely to report a worse versus a better or neutral QOL score if the Elizabethan collar had perceivable negative impacts on their pet’s ability to drink and play or if it caused injury—itching/irritation (Table 2). Brown (2006) noted that animals usually exhibit a decreased level of water consumption for a period of time after collar placement, which may coincide with a period of habituation [5]. This is supported by our finding that the majority of respondents (60.2%) reported that their pet experienced difficulties drinking while wearing the collar, and 17.1% said that their companion animal was unable to perform this activity at all while wearing the Elizabethan collar. However, we cannot determine whether animals habituated to Elizabethan collars, and over what timeframe this occurred.

Elizabethan collars can be inflexible and uncomfortable to wear [3,5,10]. When Elizabethan collars collide with furniture or walls, it can be distressing to companion animals wearing them [3,11], and, as we found, could also lead to property damage. The size and shape of Elizabethan collars may contribute to the significantly decreased level of playing and reduced interaction with other household pets which was reported by 40.3% of respondents. 

The design of the Elizabethan collars gives them the potential to cause injury to both the animal wearing it and others around them [1,4,5,20]. Of the participants who reported that their animal acquired injuries as a result of the Elizabethan collar, the majority (61.1%) of these injuries were in the form of itching/irritation. As suggested by Brown, this could be due to a sharp outer flange which can cause irritation or cuts to the limbs or ears of an animal [1], but may also have been due to difficulty in navigating their environments because of interference with ambulation, vision or hearing, or a combination of these. Other authors have reported animals vigorously shaking their heads and chewing at the collar in an attempt to remove it [4]. This behaviour may damage the collar which could be hazardous (due to sharp edges) and cause injuries, while also rending the collar ineffective [4]. Head shaking can also lead to further injuries such as aural haematomas [26]. 

This is the first report that the authors are aware of documenting injury to owners by companion animals wearing Elizabethan collars. 

### 4.1. Mitigating Welfare Costs Associated with Elizabethan Collars

Owners of animals who wore Elizabethan collars in the previous twelve months overwhelmingly reported an interest in mitigating perceived harms associated with these collars, with discussions of appropriate sizing of the collar, and judicious selection of alternatives—or in some cases a combination of alternatives—where possible.

Some authors argue that animals can habituate to Elizabethan collars over time [5], and this was certainly the experience of some of our respondents. Pre-conditioning animals to the collar before surgery may reduce post-surgical habituation time [5], but according to our respondents this was not always successful. We found that some owners reported that their animal was unable to habituate to an Elizabethan collar, which is consistent with other published reports [1,5]. To assist habituation, Brown (2006) argues that animal carers should ensure that animals are able to reach food and water when the Elizabethan collar is on, or implement an assisted feeding schedule [1]. During periods of habituation, behaviours such as rolling over, loss of balance and lying down may be seen [1]. It is vital to ensure that animals wearing collars are supervised during this habituation period [1,5,20].

The fact that a number of owners reported that their companion animal was able to access the site(s) the Elizabethan collar was being used to protect, underscores the need to observe animals closely in the period immediately following Elizabethan collar fitting. For example, if an Elizabethan collar is fitted in a veterinary hospital prior to discharge, it may be useful to observe the animal in hospital to ensure the animal is not able to access the site, and is not unduly distressed, before discharging the animal to the owner. However, as it is known that normal animal behaviour may be suppressed in veterinary clinical settings, such observations should be combined with detailed guidance for owners [21]. 

It may be possible in some cases to employ alternative methods to Elizabethan collars, which minimise negative welfare impacts including injury or misadventure [11]. Selection of an alternative depends to some degree on the indication for the Elizabethan collar. 

Physical alternatives to Elizabethan collars include inflatable collars, neck restraints, visors, muzzles, socks or booties, body wraps or clothing [11]. Pharmacological alternatives include topical and systemic anti-pruritic agents, topical and systemic analgesics, topical anaesthetic agents, anxiolytics or sedatives which may reduce the need for Elizabethan collars by preventing self-mutilation due to pain and/or anxiety [6,13]. A number of owners reported modifying the collar themselves or fashioning a collar out of household items (for example, a pool noodle). While we cannot determine whether home-made collars were more or less successful than commercially available Elizabethan collars, we believe that it would be better for owners to be aware of commercially available alternatives that have been designed to fit animals before relying on home-made Elizabethan collars, because of the potential for misadventure. 

### 4.2. Limitations

We were not able to calculate a response rate for our survey because respondents were self-selected from an unknown sampling frame. As respondents self-selected, this may have biased our sample towards those with stronger views about Elizabethan collars. In addition, a large proportion of respondents (36.4%) worked with animals. It is possible that these respondents were better equipped to deal with the potential challenges of Elizabethan collars, consider the advantages and limitations of alternative options, and be more sensitive or alert to changes in animal behaviour. Furthermore, the majority (93.5%) of respondents were female which could lead to gender bias in our results. However, this is supported by research which suggest that females are more often caregivers for pets [27]. 

As a retrospective survey this study is subject to recall bias. For example, owners were asked to complete the survey for a single animal. It is possible that in households where multiple animals wore an Elizabethan collar in the previous twelve months, that owners might respond to the survey regarding the animal that had the most positive or negative experience. 

Our question about the duration of Elizabethan collar use gave respondents options ranging from <72 h to >21 days, or intermittent use. However, a number of respondents noted in the additional comments that their animal wore the collar for very brief or very extended periods—for example, four respondents reported that their companion animal wore the collar for, at most, “a few hours”, while two respondents reported that their companion animals wore the collar continuously for three and six months, respectively. In retrospect, the options we provided reflected our own bias as veterinarians who generally see collars used for relatively short periods. Provision of an option to enter the length of time the animal wore the collar for may have revealed that more animals wore the collar for longer periods. We acknowledge that it is possible that wearing a collar for very long periods is associated with additional or welfare issues, or indeed diminished harm due to habituation, both of which are beyond the scope of the current study.

It is possible that the welfare impacts of Elizabethan collars differed between dogs and cats. Our survey did not require owners to specify the species of companion animal and therefore we were unable to determine differences between dogs and cats based on these responses. Given the wider variation of conformation in dog breeds, it may be that issues pertaining to the size and fit of Elizabethan collars are more common in dogs than cats. As our study highlighted, Elizabethan collars may have different impacts on different dog breeds due to significant variation in conformation. A larger study requiring respondents to specify the breed would be required to determine whether there is breed variation on the impact of Elizabethan collars on QOL, including risk of injury, misadventure or collar failure.

It is difficult to determine to what degree an animal’s underlying condition contributed to its poor QOL, as compared to the Elizabethan collar alone but this is likely to have been a contributing factor since Elizabethan collars are normally fitted at times when animals are suffering some sort of injury, or surgical wound. As one cat owner wrote, “It is hard to tell if the change in Snooza’s behaviours was due to the collar or because he was dying from the cancer. He was always an old snoozey cat anyway”. Nonetheless, multiple owners reported an immediate negative change in the demeanor of their animal when the Elizabethan collar was fitted, and immediate positive change in the demeanor of their animal when the Elizabethan collar was removed. Many animals wore the Elizabethan collar for treatment of conditions, such as mild dermatological conditions, which may not be expected to impact the animal’s overall demeanor.

Furthermore, there is a potential that QOL score reported by owners could be impacted by the process of selecting and fitting the Elizabethan collar. It is possible that collars fitted by a veterinary professional (e.g., veterinarian, animal health technician or veterinary nurse) may have been associated with a better-fitted collar and possibly promoted a better QOL score. However, no questions were asked about how the collar was fitted in our survey.

Because commercially available Elizabethan collars are predominantly made from rigid plastic, respondents were not given an option of selecting the type of Elizabethan collar used on their animal. We are therefore unable to determine whether different types of Elizabethan collars had different impacts on various activities or owner reported QOL. This could be explored in future studies.

### 4.3. Recommendations

1. Where appropriate, explore the impacts of alternatives further, so as to confidently recommend their use.
2. Given that the key indication for wearing Elizabethan collars was to protect surgical sites on the body, head and neck from self-trauma (accounting for 69.4% of cases), veterinary team members have an important role in counselling owners and carers of companion animals about the potential negative impacts of Elizabethan collars.
3. At a minimum, we suggest that any person recommending the use of an Elizabethan collar in a companion animal, or considering using such a collar on their own animal, should be made aware of the potential for the Elizabethan collar to interfere with activities such as drinking and playing (including playing with other animals), the potential for discomfort or injury to animals, and the potential for injury to people in the vicinity of Elizabethan collar wearing animals, as well as harm minimisation strategies.

## 5. Conclusions

Despite the ongoing use of Elizabethan collars in veterinary medicine, we have shown that they may negatively impact animal welfare and overall QOL in a range of important domains. The findings of this study suggest that an owner-perceived worse QOL outcome was more likely to be reported if the Elizabethan collar impacted the animal’s ability to drink, play or caused itching/irritation to their pet. 

Numerous alternatives to Elizabethan collars are available and the efficacy of these should be explored. Future studies could explore differences between Elizabethan collar welfare and QOL implications on dogs and cats and explore whether reduced QOL score was associated with the Elizabethan collar alone or was due to the injury/surgery that indicated the collar’s use. Future studies could also examine whether the use of better pharmacological treatment such as analgesia, anti-pruritics or anxiolytics can reduce the need for Elizabethan collars.

## Figures and Tables

**Table 1:** Characteristics of respondents in an online survey surrounding the welfare implications of Elizabethan collars on companion dogs and cats.

Demographic Information	Category	No.	%
Gender	Female	406	93.5
	Male	22	5.1
	Other	3	0.7
	(blank)	3	0.7
Location	New South Wales	183	42.2
	Queensland	39	9.0
	Victoria	37	8.5
	Western Australia	14	3.0
	South Australia	8	1.8
	Northern Territory	3	0.7
	Tasmania	5	1.2
	UK	66	15.2
	USA	55	12.7
	New Zealand	3	0.7
	South Africa	1	0.2
	Ireland	1	0.2
	Sweden	1	0.2
	(blank)	18	4.2
Age	18–19	5	1.2
	20–29	97	22.4
	30–39	108	24.9
	40–49	104	24.0
	50–59	62	14.3
	60–69	39	9.0
	70–79	12	2.8
	80–89	3	0.7
	(blank)	4	0.9
Worked with animals *	No	276	63.6
	Yes	158	36.4
Responsibility	Mostly my responsibility	310	71.4
	Mostly someone else’s responsibility	3	0.7
	Shared between household members	121	27.9

* veterinarian, veterinary nurse, veterinary technician, animal trainer or person who works with animals for a living.

**Table 2:** Factors identified as being the best predictors of a worse quality of life outcome in an online survey conducted on welfare implications of Elizabethan collar (Elizabethan collar) use in companion dogs and cats.

Variable	Category	B	SE	OR	95% CI
Constant	−	−1.469	0.589	0.2	−
Drinking ^a^	1	1.688	0.710	5.4	1.3–21.7
	2	1.762	0.587	5.8	1.8–18.4
	3	1.103	0.561	3.0	1.0–9.1
	4	2.269	0.605	9.7	3.0–31.7
	5 *	0	−	1	
Playing ^b^	1	3.831	1.168	46.1	4.7–454.9
	2	1.123	0.650	3.1	0.86–11.0
	3	0.887	0.668	2.4	0.7–9.0
	4	1.225	0.684	3.2	0.9–13.0
	5 *	0	−	1	
Injury (Itching/ Irritation) ^c^	Yes	2.466	1.087	11.8	1.4–99.0
	No*	0	−	1	

B = beta value; SE = standard error; OR = odds radio; CI = Confidence interval. ^a^ Variable(s) entered on step 1: Drinking. ^b^ Variable(s) entered on step 2: Playing. ^c^ Variable(s) entered on step 3: Did your companion animal acquire any injuries as a result of the Elizabethan collar? (tick all that apply) (choice = Itching/irritation). * Reference category.

## Appendix A

### Appendix A.1. Questionnaire

**Question**	**Options**
1. Has your cat or dog worn an Elizabethan Collar in the past twelve months?	- Yes (continue to survey) - No (Thank you for your interest. This survey is only open to persons whose dog has worn an Elizabethan collar in the past six months)
2. Your age (years) _____	*insert age in years*
3. Your gender	- Male - Female - Other
4. Postcode	*insert postcode*
5. Are you a veterinarian, veterinary nurse, veterinary technician, animal trainer or a person who works with animals for a living?	- Yes - No
6. In your household, responsibility for the care of your companion animal is:	- Mostly my responsibility - Mostly someone else’s responsibility - Shared between household members
7. How long was your companion animal required to wear an Elizabethan collar?	- < 24 h - 24–72 h - 72 h–7 days - 8–14 days - 15–21 days - > 21 days - Intermittent use - Can’t recall
8. What was the reason?	- To protect a surgical site on the body - To protect a surgical site on the head or neck - To protect a bandage/drain/implant - To prevent self-trauma because of a skin condition - Other (Please describe)
9. Which activities, if any, did the collar interfere with (rate all that apply) [1 = cannot perform this activity when wearing collar; 2 = has some difficulty performing this activity when wearing collar; 3 = needs assistance when performing this activity; 4 = Elizabethan collar makes minimal difference when performing this activity; 5 = Elizabethan collar makes no difference to performance of this activity]	- Eating 1–5 - Drinking 1–5 - Walking 1–5 - Other exercise 1–5 - Interacting with people 1–5 - Playing 1–5 - Resting/sleeping 1–5 - Outdoor access 1–5 - Other (please add) 1–5
10. Are you aware of how to check whether your companion animal Elizabethan collar is too tight/loose or not?	- Yes - No - Don’t know
11. Did your companion animal exhibit any difficulty breathing while wearing the Elizabethan collar?	- Yes - No - Not sure
10a. If yes, did these difficulties breathing exist before the use of the Elizabethan collar due to prior health problems?	- Yes (please specify) - No
12. Were you required to re-size or replace your pet’s Elizabethan collar in the duration that they were required to use it?	- Yes - No - Don’t know
11a. If yes, why?	- The collar was too tight - The collar was too loose - My dog was continuously trying to destroy the collar - Other (please specify)
13. Did your companion animal acquire any injuries as a result of the Elizabethan collar? (tick all that apply)	- Skin of the neck was abraded/ulcerated - Injury due to limbs getting caught in collar - Injury due to interactions with other pets - Itching/irritation - Other (please specify) - Not applicable
14. While wearing the collar, you would say that your pet’s response to sound is:	- As usual - More sensitive to sound - Less sensitive to sound - Not responsive to sound at all
15. Did you remove the Elizabethan collar during the time its use was prescribed:	- Never - Only for certain activities (for example eating, drinking) - Only under supervision - Most of the time
16. How often did your companion animal remove the collar without your assistance	- Never - Sometimes - Frequently - Couldn’t keep it on him/her
17. If you had any other companion animals at the time your companion animal was required to use an Elizabethan collar, did interactions between these animals change?	- Reduced interaction - Increased friendly interactions - Increased unfriendly interactions - Decreased friendly interactions - Decreased unfriendly interactions - No change in interactions - Not applicable
18.Have you ever used alternatives to achieve the same purpose as an Elizabethan collar? (tick all that apply)	- Local dressing - T-shirt or wrap - Sedation - E collar alternative such as an inflatable collar - Other (please describe) - No
17a. If so, was it:	- More successful than an Elizabethan collar? - About as effective as an Elizabethan collar - Less effective than an Elizabethan collar
19. Rate your companion animal’s quality of life WITH the Elizabethan collar from 1–5 [1 (Couldn’t be better)–5 (very poor)]	- 1 - 2 - 3 - 4 - 5
20. Rate your companion animal’s quality of life WITHOUT the Elizabethan collar from 1–5 [1 (Couldn’t be better)–5 (very poor)]	- 1 - 2 - 3 - 4 - 5
21. Do you wish to provide any additional comments?	*insert any addition comments*

**Table A1:** Responses from participants in an online survey about welfare implications of Elizabethan Collar use on companion dogs and cats. The survey was conducted in 2019 and a total of 439 responses were collected.

Question	Category	No.	%
How long was your companion animal required to wear an Elizabethan collar?	< 24 h	10	2.3
	24–72 h	61	14.1
	72 h–7 days	158	36.4
	8–14 days	114	26.3
	15–21 days	34	7.7
	> 21 days	30	6.9
	Intermittent use	24	5.5
	Can’t recall	4	0.9
What was the reason?	To protect a surgical site on the body	249	57.4
	To protect a surgical site on the head or neck	52	12.0
	To protect a bandage/drain/implant	22	5.1
	To prevent self-trauma because of a skin condition	83	19.1
	Other [Please describe]	28	6.5
Which activities, if any, did the collar interfere with (rate all that apply)			
A. Eating	1 ^a^	91	21.0
	2	120	27.6
	3	75	17.3
	4	91	21.0
	5	52	12.0
	NA	4	0.9
	(blank)	1	0.2
B. Drinking	1 ^a^	74	17.1
	2	124	28.6
	3	63	14.5
	4	110	25.3
	5	61	14.1
	NA	1	0.2
	(blank)	1	0.2
C. Walking	1 ^a^	21	4.8
	2	60	13.8
	3	72	16.6
	4	150	34.6
	5	123	28.3
	NA	6	1.4
	(blank)	2	0.5
D. Other exercise	1 ^a^	44	10.1
	2	68	15.7
	3	84	19.4
	4	113	26.0
	5	67	15.4
	NA	51	11.8
	(blank)	7	1.6
E. Interacting with people	1^a^	22	5.1
	2	87	20.0
	3	96	22.1
	4	132	30.4
	5	90	20.7
	NA	4	0.9
	(blank)	3	0.7
F. Playing	1^a^	83	19.1
	2	125	28.8
	3	85	19.6
	4	69	15.9
	5	38	8.8
	NA	32	7.3
	(blank)	2	0.5
G. Resting/sleeping	1^a^	30	6.9
	2	82	18.9
	3	70	16.1
	4	145	33.4
	5	104	24.0
	NA	2	0.5
	(blank)	1	0.2
H. Outdoor access	1^a^	59	13.6
	2	67	15.4
	3	90	20.7
	4	74	17.1
	5	95	21.9
	NA	44	10.1
	(blank)	5	1.2
I. Other [please add]	Other	179	41.2
Are you aware of how to check whether your companion animal’s Elizabethan collar is too tight/loose?	Yes	382	88.0
	No	39	9.0
	Don’t know	13	3.0
Did your companion animal exhibit any difficulty breathing while wearing the Elizabethan collar?	Yes	3	0.7
	No	426	98.2
	Not sure	5	1.2
Were you required to re-size or replace your pet’s Elizabethan collar in the duration that they were required to use it?	Yes	122	28.1
	No	306	70.5
	Don’t know	2	0.5
	(blank)	4	0.9
Did your companion animal acquire any injuries as a result of the Elizabethan collar (tick all that apply)	Skin of the neck was abraded/ulcerated	14	3.2
	Injury due to limbs getting caught in collar	2	0.5
	Injury due to interaction with other pets	1	0.2
	Itching/irritation	69	15.9
	Other	27	6.2
	NA	328	75.6
While wearing the collar, you would say that your companion animal’s response to sound is:	As usual	299	68.9
	Less sensitive to sound	50	11.5
	More sensitive to sound	84	19.4
	Not responsive to sound at all	1	0.2
Did you remove the Elizabethan collar during the time its use was prescribed?	Most of the time	44	10.1
	Never	47	10.8
	Only for certain activities (for example eating, drinking)	108	24.9
	Only under supervision	235	54.1
How often did your companion animal remove the collar without your assistance?	Never	299	68.9
	Sometimes	103	23.7
	Frequently	21	4.8
	Couldn’t keep it on him/her	11	2.5
If you had any other companion animals at the time your companion animal was required to use an Elizabethan collar, did interactions between these animal’s change?	Reduced interaction	146	33.6
	Increased friendly interactions	10	2.3
	Increased unfriendly interactions	38	8.8
	Decreased friendly interactions	27	6.2
	Decreased unfriendly interactions	2	0.5
	No change in interactions	90	20.7
	Not applicable	121	27.9
Have you ever used alternatives to achieve the same purpose? (tick all that apply)	Local Dressing	76	17.5
	T-shirt or wrap	105	24.2
	Sedation	19	4.4
	Elizabethan collar alternative, e.g., inflatable collar	120	27.6
	Other	41	9.4
	No	209	48.2
Rate your companion animal’s quality of life WITH the Elizabethan collar from 1-5	1 ^b^	32	7.4
	2	107	24.7
	3	135	31.1
	4	107	24.7
	5	51	11.8
	(blank)	2	0.5
Rate your companion animal’s quality of life WITHOUT the Elizabethan collar from 1-5	1 ^b^	305	70.3
	2	57	13.1
	3	14	3.2
	4	21	4.8
	5	35	8.1
	(blank)	2	0.5

a—1 (cannot perform this activity when wearing Elizabethan collar); 2 (has some difficulty performing this activity when wearing Elizabethan collar); 3 (needs assistance when performing this activity); 4 (Elizabethan collar makes minimal difference when performing this activity); 5 (Elizabethan collar makes no difference to performance of this activity); NA (not applicable); b—1 (couldn’t be better); 5 (very poor).

**Table A2:** Chi-square univariate analysis of responses from participants in an online survey of welfare implications of Elizabethan Collar use on companion dogs and cats, conducted in 2019. Predictor variables were screened for an association with the outcome (better, neutral or worse quality of life score). Significant variables in bold (*p* < 0.2).

Variable/Question	Category	No.			Chi Squared	*p* Value
		Better	Neutral	Worse		
How long was your companion animal required to wear an Elizabethan collar?	< 72 h	6	2	63	17.2	0.069
	72 h–7 days	23	13	122		
	8–14 days	13	17	84		
	15–21 days	5	6	22		
	> 21 days	5	0	25		
	Intermittent use	4	3	17		
What was the reason?	To protect a surgical site on the body	27	24	198	7.9	0.446
	To protect a surgical site on the head or neck	7	6	39		
	To protect a bandage/drain/implant	2	1	19		
	To prevent self-trauma because of a skin condition	16	6	61		
	Other [Please describe]	4	5	19		
Which activities, if any, did the collar interfere with (rate all that apply)						
A. Eating	1	7	4	80	55.3	<0.001
	2	11	4	105		
	3	11	6	58		
	4	11	12	68		
	5	14	16	22		
B. Drinking	1	5	2	67	62.1	<0.001
	2	7	5	112		
	3	13	6	44		
	4	14	11	85		
	5	15	18	28		
C. Walking	1	0	1	20	21.5	0.006
	2	7	1	52		
	3	8	5	59		
	4	19	13	118		
	5	20	22	81		
D. Other exercise	1	2	1	41	28.5	<0.001
	2	7	4	57		
	3	10	9	65		
	4	20	8	85		
	5	7	17	43		
E. Interacting with people	1	2	1	19	19.3	0.014
	2	8	2	77		
	3	12	8	76		
	4	17	14	101		
	5	15	17	58		
F. Playing	1	5	1	77	41.6	<0.001
	2	11	9	105		
	3	12	6	67		
	4	10	12	47		
	5	11	9	18		
G. Resting/sleeping	1	3	1	26	17.8	0.023
	2	7	2	73		
	3	8	5	57		
	4	22	15	108		
	5	14	18	72		
H. Outdoor access	1	5	0	54	23.1	0.003
	2	8	4	55		
	3	10	6	74		
	4	15	9	50		
	5	13	17	65		
Are you aware of how to check whether your companion animal’s Elizabethan collar is too tight/loose?	Yes	48	42	292	4.8	0.090
	No	5	0	34		
Did your companion animal exhibit any difficulty breathing while wearing the Elizabethan collar?	Yes	0	0	3	0.9	0.639
	No	56	42	328		
Were you required to re-size or replace your pet’s Elizabethan collar in the duration that they were required to use it?	Yes	19	7	96	3.6	0.168
	No	35	33	238		
Did your companion animal acquire any injuries as a result of the Elizabethan collar (tick all that apply)						
A. Skin of the neck was abraded/ulcerated	Yes	2	2	10	0.406	0.816
	No	54	40	326		
B. Injury due to limbs getting caught in collar	Yes	0	0	2	0.586	0.746
	No	56	42	334		
C. Injury due to interaction with other pets	Yes	0	0	1	0.292	0.864
	No	56	42	335		
D. Itching/irritation	Yes	4	3	62	7.258	0.027
	No	52	39	274		
E. Other	Yes	1	2	24	2.531	0.282
	No	55	40	312		
While wearing the collar, you would say that your companion animal’s response to sound is:	As usual	40	36	223	7.807	0.099
	Less sensitive to sound	6	4	41		
	More sensitive to sound	10	2	72		
Did you remove the Elizabethan collar during the time its use was prescribed?	Most of the time	1	2	41	13.612	0.034
	Never	6	9	32		
	Only for certain activities (for example eating, drinking)	14	7	87		
	Only under supervision	35	24	176		
How often did your companion animal remove the collar without your assistance?	Never	41	28	230	3.139	0.535
	Sometimes	11	13	79		
	Frequently	4	1	27		
If you had any other companion animals at the time your companion animal was required to use an Elizabethan collar, did interactions between these animal’s change?	Decreased	24	12	139	15.5328	0.004
	Increased	7	1	40		
	No change in interactions	12	18	60		
Have you ever used alternatives to achieve the same purpose? (tick all that apply)						
A. Local Dressing	Yes	9	9	58	0.541	0.763
	No	47	33	278		
B. T-shirt or wrap	Yes	13	8	84	0.755	0.686
	No	43	34	252		
C. Sedation	Yes	3	3	13	1.103	0.576
	No	53	39	323		
D. Elizabethan collar alternative	Yes	18	9	93	1.378	0.502
	No	38	33	243		
E. Other	Yes	3	6	32	2.247	0.325
	No	53	36	304		

*Responses ‘don’t know’, ‘not sure’, ‘can’t recall’, ‘NA/not applicable’ were labelled as missing for data analysis P value of significant variables are bolded.
